# Regulation of Conidiation and Aflatoxin B1 Biosynthesis by a Blue Light Sensor LreA in *Aspergillus flavus*

**DOI:** 10.3390/jof10090650

**Published:** 2024-09-13

**Authors:** Kunzhi Jia, Yipu Jia, Qianhua Zeng, Zhaoqi Yan, Shihua Wang

**Affiliations:** Key Laboratory of Pathogenic Fungi and Mycotoxins of Fujian Province, Key Laboratory of Biopesticide and Chemical Biology of Education Ministry, School of Life Sciences, Fujian Agriculture and Forestry University, Fuzhou 350002, China; kjia@fafu.edu.cn (K.J.); 15032034086@126.com (Y.J.); 3210537002@fafu.edu.cn (Q.Z.); 2023027305@stu.sdnu.edu.cn (Z.Y.)

**Keywords:** *Aspergillus flavus*, aflatoxin B1, conidiation, LreA

## Abstract

Conidia are important for the dispersal of *Aspergillus flavus*, which usually generates aflatoxin B1 (AFB1) and poses a threat to the safety of agricultural food. The development of conidia is usually susceptible to changes in environmental conditions, such as nutritional status and light. However, how the light signal is involved in the conidiation in *A. flavus* is still unknown. In this study, LreA was identified to respond to blue light and mediate the promotion of conidiation in *A. flavus*, which is related to the central development pathway. At the same time, blue light inhibited the biosynthesis of AFB1, which was mediated by LreA and attributed to the transcriptional regulation of *aflR* and *aflS* expression. Our findings disclosed the function and mechanism of the blue light sensor LreA in regulating conidiation and AFB1 biosynthesis, which is beneficial for the prevention and control of *A. flavus* and mycotoxins.

## 1. Introduction

Fungi perceive light, which plays a crucial role in various biological processes, such as morphology construction and circadian rhythm [1], as environmental information, and they adjust their behaviors for improved survival [2]. In *Aspergillus nidulans*, the VelB/VeA/LaeA complex has been identified as a key player in coordinating light signals with fungal development and secondary metabolism [3]. The white collar (WC) proteins sense the bule light and regulate the circadian clock system in *Neurospora crassa* [4]. Different fungi sense light of varying colors through a range of photoreceptors. For instance, in *N. crassa*, WC proteins and cryptochromes respond to blue light, while opsins and phytochromes detect green and red light, respectively [1,2]. As a sensor of blue light in fungi, the WC proteins, typically comprised of WC1 and WC2, form a heterodimer complex known as the WCC, which is responsible for activating the transcription of light-responsive genes [5]. The transcriptional activity of WCC is modulated by the small LOV domain-containing protein vivid (VVD) through direct interactions, a mechanism observed in many fungal species [1]. In *A. nidulans*, LreA and LreB, analogous to WC-1 and WC-2, respectively, serve as blue light sensors [6]. These proteins likely form a complex with similar transcriptional activity as the WCC. Notably, the red light sensor FphA is involved in the transcriptional complex associated with WCC in this fungal species [6]. Other fungi, such as *Phycomyces blakesleeanus* and *Mucor circinelloides*, exhibit multiple varieties of WC orthologs with structurally similar but functionally distinct roles [2]. This diversity in blue light receptors among fungi highlights the evolutionary adaptation of these organisms, showcasing variability in molecular mechanisms across different species.

The fungus *Aspergillus flavus* is known for infecting oil crop seeds and producing aflatoxin B1 (AFB1) [7,8], which is able to cause poisoning and carcinogenesis, posing serious health risks to animals and humans [9,10]. Therefore, it is crucial for control of the fungus and aflatoxins (AFs) to investigate the growth patterns and mechanisms of mycotoxin biosynthesis in *A. flavus*. Light is important for the growth and metabolism of fungi, but the relationship of *A. flavus* and light is not described in previous research. In *A. nidulans*, blue light was shown to inhibit the biosynthesis of sterigmatocystin (ST), a precursor of AFB1 [6]. This led us to explore whether blue light plays a role in AFB1 biosynthesis in *A. flavus*. In this study, we aim to identify and functionally characterize the blue light sensor in *A. flavus*.

## 2. Materials and Methods

### 2.1. Light Source

The study utilized a blue light source with a 450 nm wavelength at an intensity below 100 LX [11]. To mitigate external light interference, the incubator was uniformly covered with shade material, maintaining a temperature of 29 °C unless stated otherwise [12].

### 2.2. Strains

All of the *Aspergillus flavus* strains used in this study are listed in Table 1.

### 2.3. Domain Structure and Phylogenetic Tree Analysis

The protein sequences of putative blue light receptors from *A. flavus*, *A. oryzae*, *A. parasiticus*, *A. tamarii*, *A. bombycis*, *A. niger*, *A. fumigatus*, and *A. nidulans* were aligned to protein sequences in databases using Blast. The phylogenetic tree was drawn using MEGA 7.0 software, and the domains analysis and domain alignment were performed with SMART (https://smart.embl.de/, accessed on 15 October 2023) and DOG 2.0 (http://dog.biocuckoo.org/, accessed on 15 October 2023), respectively.

### 2.4. Construction of Mutant Strains

The PCR primers are listed in Table A1. The knockout mutant strains for *lreA* and *lreB* (Δ*lreA* and Δ*lreB*) were constructed through homologous recombination using the *pyrG* gene (from *A. fumigatus*) to replace the *lreA* or *lreB* gene [13]. Briefly, the upstream and downstream fragments of the target gene were used to construct the fusion fragment using fusion PCR, and then the fusion fragment was transformed into the *pyrG*-deficient *A. flavus* CA14 PTS strain [14]. Positive transformants were screened through PCR. The strategy for the construction of the complementary strains (*lreA*-com and *lreB*-com) was similar to that for the knockout strain, in which gene knock protoplast was used as a recipient strain in transformation. Deletion of the mutant strain for both genes (Δ*lreA*Δ*lreB*) was developed on the basis of the Δ*lreA* strain by employing a similar single-gene knockout strategy with pyrithiamine resistance (*prtA*) as a screening marker for the knockout of a second gene. For the PAS mutant (*lreA*^ΔPAS-A^), fusion PCR was used to construct the DNA fragment by connecting the coding region of the PAS mutant and *pyrG*. Then, genomic *lreA* was replaced with the DNA fragment using homology recombination, as described above. The positive transformants were screened through PCR.

### 2.5. Mycelial Growth and Conidia Analysis

The conidia of 1 × 10^7^/mL were inoculated and cultured on different media for 3 days to observe the mycelial growth of *A. flavus*, and the cell-counting chamber was used for spore counting. Each experiment was repeated at least 3 times [12,13].

### 2.6. Aflatoxin Analysis

To produce aflatoxins (AFs), 10 μL of conidial suspension (1 × 10^7^/mL) of all of the tested *A. flavus* strains was separately inoculated into potato dextrose agar (PDA) medium plated with cellophane for 7 days. The whole mycelia were weighed after scraping and drying and then subjected to liquid nitrogen grinding, and the medium was mashed evenly. Chloroform was used for the extraction of AFs from mycelia and the culture medium. Then, 5.0 μL of the AF suspension was loaded into a silica gel plate and separated through chromatography using acetone: chloroform (1:9, V:V). The AFs were detected through thin-layer chromatography (TLC) (Haiyang chemical, Qingdao, China), and toxin production was semi-quantified through densitometric analysis [12,13].

### 2.7. Seed Infections

To evaluate the pathogenicity of *A. flavus* on plant seeds, peanut seeds were infected by soaking them in 1 × 10^7^/mL spore suspension for 30 min, and then they were placed in Petri dishes spread with moist sterile filter paper for 7 days. The infected seeds were photographed and collected for spore counting.

### 2.8. qRT-PCR

Total RNA was extracted from filaments of *A. flavus* on PDA using an RNA Extraction Kit (Tianmer biotechnology, Beijing, China). qRT-PCR reactions were performed with a pikoreal 96 real-time PCR system using SYBR Green Supermix (Takara, Kusatsu, Japan). The 2^−ΔΔCT^ method was used to quantify the expression level of the target gene [15,16,17]. qRT-PCR primers are shown in Table A2.

### 2.9. 3-D Modeling of LreA and LreA Mutant

LreA (RMZ42427.1) and LreA^ΔPAS-A^ (absence of 281–348 AA) sequences were input into the box of the website (https://swissmodel.expasy.org/interactive/, accessed on 23 November 2023). A0A7G5JEM3.1 from the AlphaFold database was used as the template for 3D modeling.

### 2.10. Prediction of Potential Function Partners

The protein–protein interaction network was built using STRING version 12.0 (https://string-db.org/, accessed on 15 December 2023). The putative LreA (AFLA_103610) was used as the query protein. The potential partners with an interaction score higher than 0.6 were kept in the interaction network, which is summarized in Table 2.

### 2.11. Statistical Analysis

GraphPad Prism 8 (GraphPad Software, San Diego, CA, USA) was used to analyze the statistics and the significance. Student’s *t* test was performed for the comparison of two different groups, while multiple group comparisons were carried out using the one-way analysis of variance (ANOVA) test.

## 3. Results

### 3.1. Identification and Analysis of LreA in A. flavus

**Figure 1:**
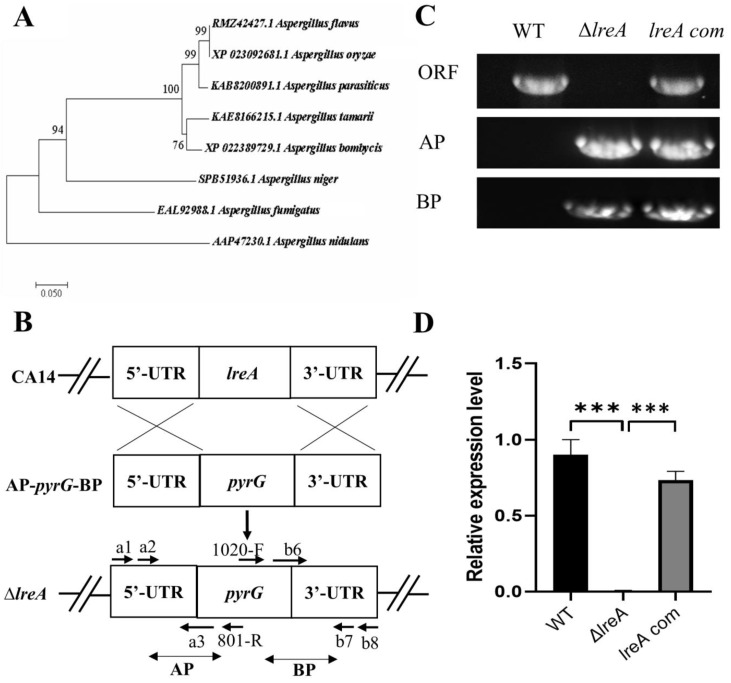
LreA identification and mutant strain construction. (**A**) Phylogenetic tree of LreA homologous proteins from various fungi. The phylogenetic tree was constructed using MEGA 7.0 with protein sequences, as shown. (**B**) A typical schematic description of *lreA* disruption. UTR represents the untranslation region. AP and BP represent the A and B homology arm parts, respectively. (**C**) *lreA* mutant strains were verified with PCR. WT means wild-type, and ORF represents the open reading frame of the *lreA* gene. (**D**) The expression of *lreA* was determined through qRT-PCR in different *A. flavus* strains. *** represents significant difference (*p* < 0.001).

To identify LreA in *A. flavus*, the protein candidate (GATA transcription factor LreA, RMZ42427.1) was found through the blast search with LreA (AAP47230.1) of *Aspergillus nidulans*, which was previously defined as the blue light receptor [6]. The alignment results showed that RMZ42427.1 was a homology of LreA (AAP47230.1) in *A. nidulans* with 63% similarity, suggesting that RMZ42427.1 (LreA) possibly mediates the function of blue light in *A. flavus*. Further alignment indicated that LreA has a high sequence similarity among *A. oryzae* (XP_023092681.1) and *A. parasiticus* (KAB8200891.1), suggesting that they may have a similar function (Figure 1A). To further study the function of LreA, the *lreA* knockout strain (Δ*lreA*) and the complementary strain (*lreA*-com) were constructed using the homology recombination method (Figure 1B), and the mutant strains were verified using the PCR method. As in Figure 1C, Δ*lreA* and *lreA*-com strains were tested using the specific primers pair, and the result showed that the homolog arms have been successfully reconstructed. At the same time, compared with the control strains (WT and *lreA*-com), Δ*lreA* has lost the open reading frame of the *lreA* gene (Figure 1C). The expression levels of *lreA* responded to the mutant strains, as expected (Figure 1D), indicating that *lreA* mutant strains were successfully constructed, which were applicable for further study.

### 3.2. LreA Promotes Conidiation by Inducing the Expression of brlA and abaA

**Figure 2:**
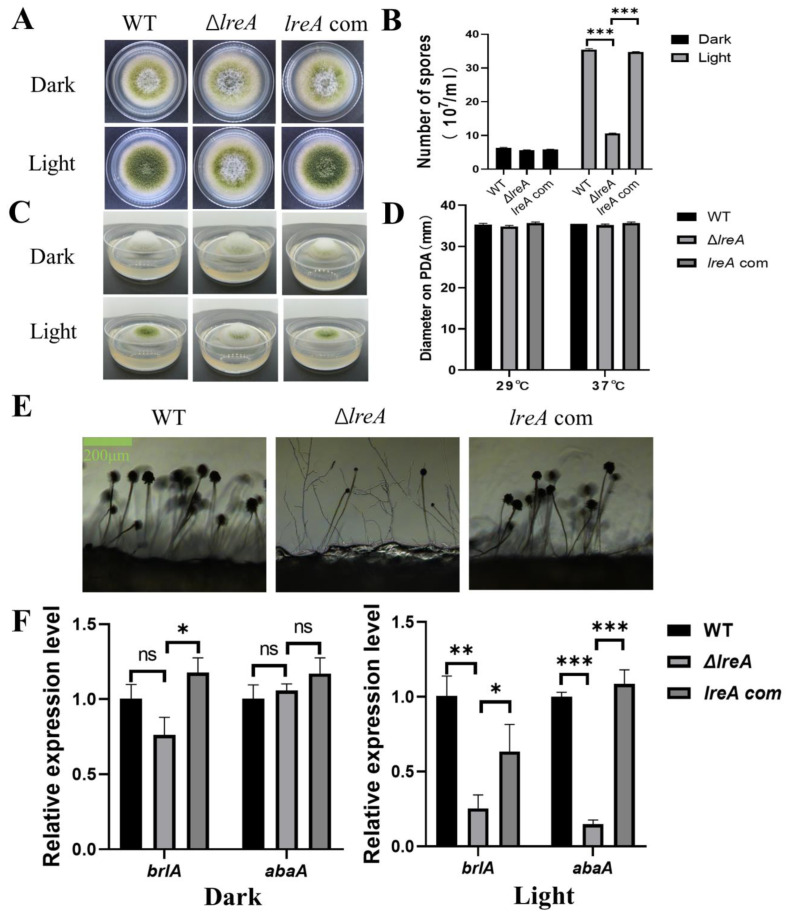
The role of *lreA* in conidiation and hyphal growth. (**A**) The growth morphology of WT, Δ*lreA,* and *lreA*-com strains on PDA. (**B**) The comparison of the conidia number in WT, Δ*lreA,* and *lreA*-com. *** represents significant difference (*p* < 0.001). (**C**) The side observation of WT, Δ*lreA,* and *lreA*-com strains’ growth on PDA. (**D**) The comparison of substrate mycelium growth in WT, Δ*lreA,* and *lreA*-com. (**E**) Conidiophore morphology of WT, Δ*lreA,* and *lreA*-com on PDA. (**F**) The expression of *brlA* and *abaA* in WT, Δ*lreA,* and *lreA*-com. * represents significant difference (*p* < 0.05), ** represents significant difference (*p* < 0.01), and *** represents significant difference (*p* < 0.001). ns, no significance.

To study the role of LreA in conidia development, conidiation was investigated in Δ*lreA* and the control strains. As shown in Figure 2A, increased conidia were observed under blue light against dark conditions in the WT strain, indicating that blue light is able to promote conidia development (Figure 2A,B). The effect of blue light on conidia development became weaker in Δ*lreA* than in the control strains (Figure 2B). The blue light inhibited the aerial mycelium in control strains, which disappeared in Δ*lreA* (Figure 2C), suggesting that LreA is necessary for the inhibition of blue light in *A. flavus*. At the same time, the blue light has no effect on the growth of substrate mycelium defined with diameter measurements (Figure 2D). At the same time, poor conidiophore and few conidia were both observed in Δ*lreA* (Figure 2E), suggesting that the promotion of conidiogenesis by blue light is mediated by LreA. As *brlA* and *abaA* genes play a critical role in the development of conidia and conidiophore [18], the gene expression of *brlA* and *abaA* has been examined in various *lreA* mutant strains. As shown in Figure 2F, the expression of *brlA* and *abaA* was significantly decreased in Δ*lreA* when compared with WT and *lreA*-com, especially in the blue light. These data suggested that the absence of LreA caused the decreased expression of *brlA* and *abaA*, subsequently impairing the development of conidia and conidiophores in Δ*lreA*.

### 3.3. LreA Inhibits AFB1 Biosynthesis by Downregulating the Expression of Key Genes

**Figure 3:**
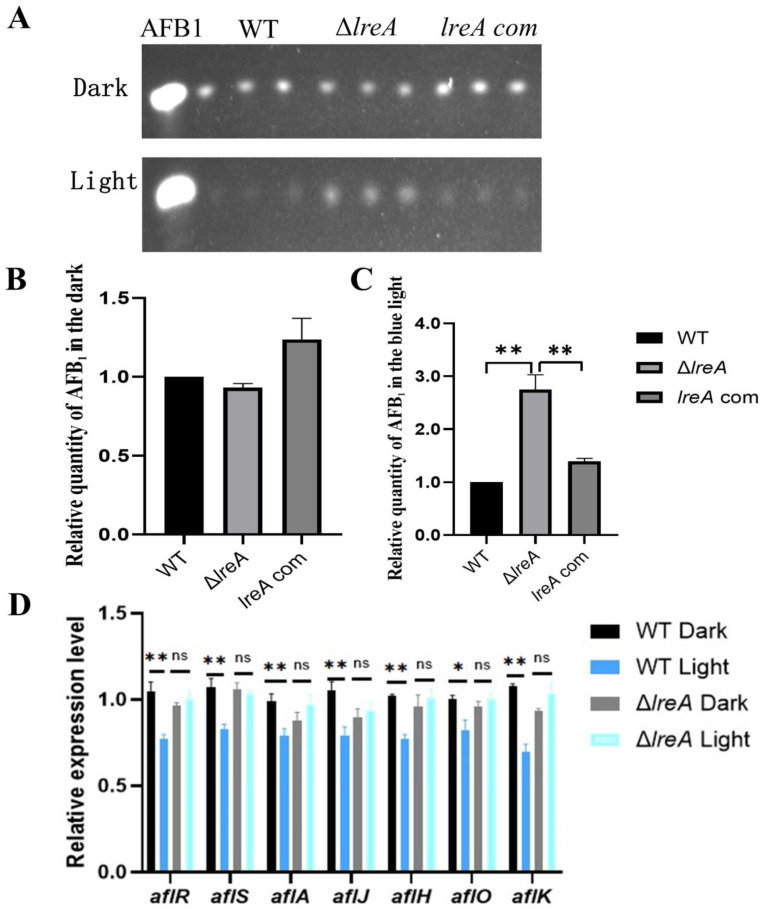
LreA was involved in biosynthesis of AFB1. (**A**) TLC analysis of AFB1 in WT, Δ*lreA,* and *lreA*-com. (**B**) The relative quantity of AFB1 in WT, Δ*lreA*, and *lreA*-com in the dark. (**C**) The relative quantity of AFB1 in WT, Δ*lreA*, and *lreA*-com in the blue light. The symbol (**) represents significant difference (*p* < 0.01). (**D**) The relative expression level of related genes in AFB1 biosynthesis cluster (*, *p* < 0.05; **, *p *< 0.01). ns, no significance.

To study the role of LreA in the biosynthesis of AFB1, aflatoxin levels were assayed in Δ*lreA* and control strains (WT and *lreA*-com) under dark and light conditions. As shown in Figure 3A,B, no significant difference was detected in Δ*lreA* or control strains in the dark. In contrast, the levels of AFB1 in Δ*lreA* were significantly higher than in the WT strain in the blue light (Figure 3C), indicating that LreA inhibited AFB1 synthesis in the blue light, which suggested that the inhibiting signal of blue light had not been sensed in Δ*lreA*. To further investigate the reason why AFB1 decreased in Δ*lreA*, the expression of key genes in AFB1 biosynthesis was examined using qPCR. As shown in Figure 3D, compared to the dark condition, the expression levels of genes, such as *aflA*, *aflJ*, *aflH*, *aflO*, and *aflK,* involved in AFB1 biosynthesis were significantly decreased in the WT strain under blue light, indicating that decreased AFB1 levels were due to the inhibited expression of AFB1 biosynthesis genes under light. In contrast, this decrease in AFB1 levels and related genes was not observed in Δ*lreA* (Figure 3D). These data suggested that LreA was responsible for sensing the blue light and mediated the inhibiting effect of AFB1 biosynthesis by downregulating the key genes for AFB1 biosynthesis in *A. flavus*.

### 3.4. PAS (Per-ARNT-Sim) Domain Is Necessary for the Function of LreA

**Figure 4:**
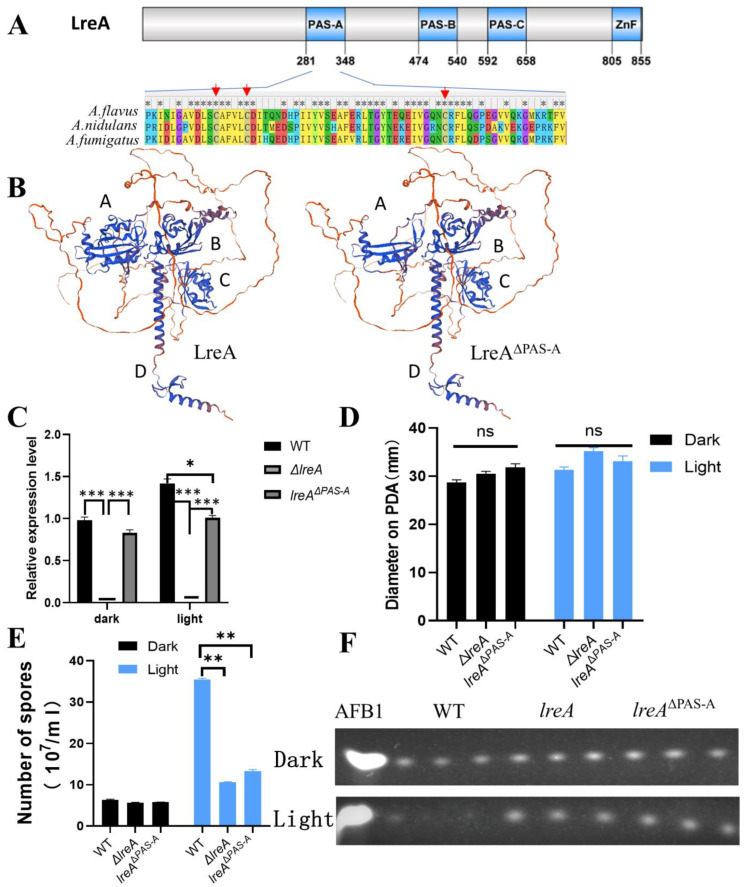
PAS domain is indispensable for the intact function of LreA. (**A**) Scheme of *A. flavus* LreA protein. An asterisk (*) indicates positions which have a single, fully conserved residue. The conserved cysteines are marked with red arrows. (**B**) The 3D modeling of LreA and LreA mutant structure. Different letters (A, B, C, and D) represent the typical domains of spatial structure. (**C**) The relative expression of *lreA* in WT and *lreA*-com or *lreA* mutant in Δ*lreA*. The symbol (*) represents significant difference (*, *p* < 0.05; ***, *p* < 0.001). (**D**) The comparison of hyphal growth in WT, Δ*lreA,* and *lreA*^ΔPAS-A^. ns, no significance. (**E**) The comparison of conidia number in WT, Δ*lreA,* and *lreA*^ΔPAS-A^ (**, *p* < 0.01). (**F**) TLC analysis of AFB1 in WT, Δ*lreA*, and *lreA*^ΔPAS-A^.

LreA mainly contains three PAS domains (PAS-A, PAS-B, PAS-C) and a zinc finger (ZnF) domain with protein domain prediction (Figure 4A). PAS-A was believed to be responsible for the binding of flavins and for sensing the light signal [1,6]. Alignment of PAS-A (281–348 AA) indicated that the similarity of PAS-A from *A. flavus* is 82% and 88% compared to that from *A. nidulans* and *A. fumigatus,* respectively, indicating that PAS-A is conserved during LreA evolution. The cysteines, which were candidates to form adducts with flavins [2,19], and the neighboring amino acids were conserved in three blue light receptors of *A. flavus*, *A. nidulans,* and *A. fumigatus* (Figure 4A). Four typical domains (A, B, C, and D) were clearly observed in the structure modeling (Figure 4B), and the absence of PAS-A disrupts the structure of domain A, which may abort the function of LreA. To explore the function of the PAS-A domain, a PAS mutant (*lreA*^ΔPAS-A^) was constructed in this study, which was confirmed with PCR and specific region sequencing in the genome. Interestingly, the expression of *lreA* was stimulated under blue light in the WT strain, while *lreA*^ΔPAS-A^ lost the ability to respond to blue light (Figure 4C). Compared with the WT strain, the blue light has no effect on the growth of substrate mycelium in *lreA*^ΔPAS-A^ (Figure 4D), but it impaired the development of conidia under light (Figure 4E), which was consistent with the phenomenon in Δ*lreA*. At the same time, the aflatoxin level was decreased in the WT due to the inhibition by blue light, but the inhibited effect of light on aflatoxin biosynthesis was relieved in *lreA*^ΔPAS-A^ (Figure 4F), which was similar to Δ*lreA*, indicating that the PAS-A domain was necessary for the normal role of LreA in *A. flavus*.

### 3.5. LreB Functions as a Potential Partner of LreA in A. flavus

**Figure 5:**
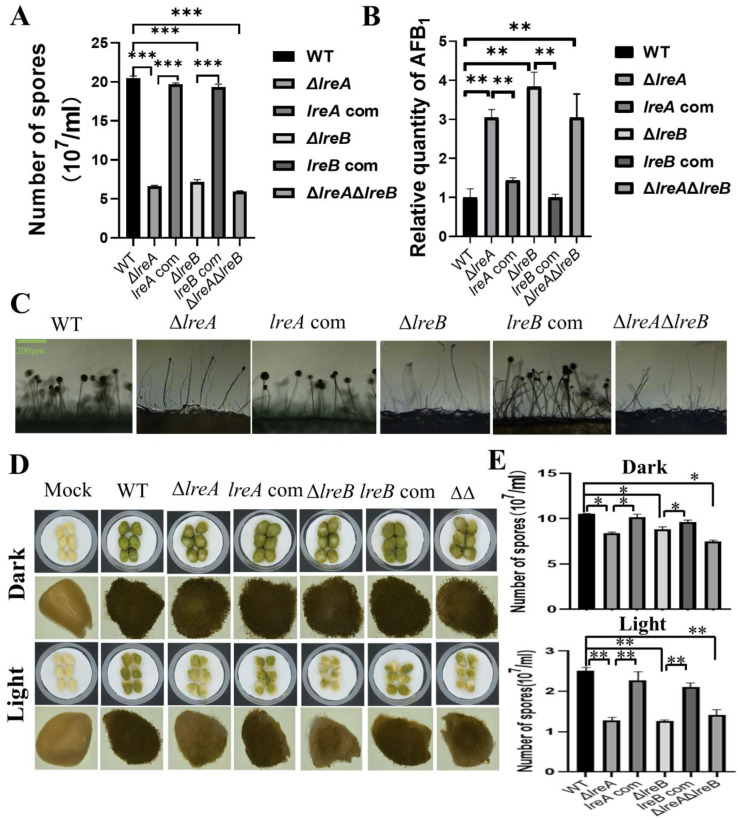
LreB functions as a potential partner of LreA in *A. flavus*. (**A**) The comparison of conidia number in WT, Δ*lreA*, *lreA*-com, Δ*lreB*, *lreB*-com, and Δ*lreA*Δ*lreB* grown in the blue light (***, *p* < 0.001). (**B**) The relative quantity of AFB1 in WT, Δ*lreA*, *lreA*-com, Δ*lreB*, *lreB*-com, and Δ*lreA*Δ*lreB* grown in the blue light (**, *p* < 0.01). (**C**) Conidiophore morphology of WT, Δ*lreA*, *lreA*-com, Δ*lreB*, *lreB*-com, and Δ*lreA*Δ*lreB*. (**D**) Infection of peanuts with various strains of *A. flavus*. Mock represents a control without any infection. (**E**) The comparison of conidia number in infected peanuts with various strains of *A. flavus* (*, *p* < 0.05; **, *p* < 0.01).

To further characterize LreA, five proteins have been predicted to interact with LreA (Table 2). The potential LreA partners with high confidence are AFLA_065850 and AFLA_051690 (Table 2), which are homologs of FphA and LreB, respectively, in *A. nidulans*. LreB has been reported to be the transcriptional partner of LreA, and it co-regulates the expression of many downstream genes in *A. nidulans* and *N. crassa* [6]. To disclose whether LreB is involved in the regulation of conidiation and AFB1 biosynthesis in *A. flavus*, Δ*lreB* was constructed through the same method used to construct Δ*lreA*, as described above. As shown in Figure 5A, abnormal development of conidia was observed in Δ*lreB*, as in Δ*lreA*. Moreover, the absence of LreB impaired the inhibiting effect of blue light on AFB1 biosynthesis (Figure 5B), suggesting that LreB is necessary for the intact effect of blue light on *A. flavus* mediated by LreA. The Δ*lreA*Δ*lreB* double mutant confirmed the phenotype observed in Δ*lreA* and Δ*lreB* (Figure 5A–C). These results demonstrated that LreB and LreA potentially co-regulated conidiation and AFB1 biosynthesis in *A. flavus*.

Considering that *A. flavus* widely contaminates oil crop seeds, including peanut, corn, and rice, the pathogenicity of *A. flavus* was assayed through the infection of peanuts with various *A. flavus* strains in this study. As shown in Figure 5D, compared with control strain, the growth of *A. flavus* in infected peanuts was observably decreased in Δ*lreA*, Δ*lreB*, and Δ*lreA*Δ*lreB*. Correspondingly, the conidia in Δ*lreA*, Δ*lreB*, and Δ*lreA*Δ*lreB* were significantly fewer than in the WT strain (Figure 5E). These results indicated that the absence of LreA and LreB impaired the pathogenicity of *A. flavus*.

## 4. Discussion

RMZ42427.1 was identified as a blue light sensor LreA in *A. flavus*, which has 63% similarity with LreA of *A. nidulans* and contains typical domains, such as PAS and ZnF, conserved in blue light receptors [20]. The alignment of PAS domain proteins indicated that blue light receptors may extensively exist in Aspergillus fungi (Figure 1A), for which the function was still undefined, except for in *A. nidulans* and *A. fumigatus*. The high similarity of PAS-containing proteins between *A. flavus* and *A. oryzae* or *A. parasiticus* hints at their functional similarity. To study the function of LreA, Δ*lreA* and *lreA*-com mutants were successfully constructed. Our results confirmed that LreA mediated the observed function of blue light in *A. flavus*, indicating that LreA is a blue light sensor and indispensable for the blue light effect observed. Similarly, other blue sensors also exist in *A. flavus*, such as LreB, which have been reported in other fungi [1,21].

Our study indicated that LreA is involved in the development of conidia, which is the important form for *A. flavus* dispersal [18]. Given conidiophore is the specialized structure for conidia development [18,22], the poor conidiophores in Δ*lreA* reasonably failed to provide the proper microenvironment for conidia and impaired the conidiation (Figure 2C), which is consistent with previous observations [13,23]. In addition, deficient expression of *brlA* and *abaA* was indicated in Δ*lreA* (Figure 2D,E). Considering that conidia development is mainly regulated by the central development BrlA-AbaA-WetA pathway [18,24,25], this impairment of conidiation is possibly due to the poor conidiophores and the deficiency of BrlA and AbaA. Moreover, BrlA is necessary and sufficient for conidiophore development [26,27,28]. Therefore, transcriptional regulation of *brlA* plays a crucial role in the development of conidia in Δ*lreA* (Figure 2D). How LreA regulates the expression of *brlA* remains unknown and is worthy of further investigation.

Blue light was revealed to inhibit the biosynthesis of AFB1. In *A. nidulans*, white light and blue light have been indicated to inhibit ST production, in which VeA is reported to play a role in the important precursor of AFB1 biosynthesis [3,6]. Thus, the inhibition of AFB1 was possibly due to the ST inhibition in *A. flavus*. The biosynthesis of AFB1 was composed of more than 25 enzymatic reactions [29,30], and these enzymes were encoded by genes located at the AF biosynthesis gene cluster and regulated by the master regulators AflR and AflS [30,31]. In this study, the expressions of *aflR* and *aflS* were significantly decreased under blue light (Figure 3C). Thus, as the target genes of *aflR* and *aflS*, *aflA*, *aflJ*, *aflH*, *aflO*, and *aflK* were decreasingly expressed (Figure 3C), which was responsible for the biosynthesis of ST and AFB1. Therefore, LreA mediated the inhibiting effect of blue light on the biosynthesis of AFB1 by depressing the expression of *aflR* and *aflS*.

LreA was composed of four predicted domains, including three PAS domains (PAS-A, PAS-B, and PAS-C) and the ZnF domain (Figure 4A). PAS domains are essential for protein interaction and sensing environmental stimuli [32]. In *A. flavus*, the PAS-A sequence is highly conserved relative to the whole protein sequence of LreA (Figure 4A), suggesting that PAS-A plays an important role in the intact function of LreA. Thus, the absence of PAS-A destroyed domain A, a critical pocket for the cofactor flavins’ binding (Figure 4B) [33,34], which consequently impaired the interaction between LreA and the potential transcriptional partner [2,32,35]. The importance of PAS-A was further confirmed by the fact that the absence of PAS-A aborted the observed function of LreA in the conidiation and AFB1 biosynthesis (Figure 4D,E). These data demonstrated that the PAS-A domain is indispensable for the function of LreA.

Our data also suggested that the role of LreA in conidiation and AFB1 biosynthesis is attributable to the transcriptional regulation of key genes in *A. flavus*. The absence of LreB, as the transcription partner, was found to share a similar phenotype to Δ*lreA* (Figure 5A,B), which was also observed in *Beauveria bassiana* [36]. LreA and LreB tend to constitute a hetero-protein complex and regulate the transcription of downstream genes [2,37]. Thus, transcription regulation is speculated to be the main way LreA is involved in conidiation and AFB1 biosynthesis, which is strengthened by the fact that the interacting proteins of LreA predicted in this study were mainly transcriptional factors (Figure 5A). Our data demonstrated that LreA and transcriptional partner LreB mediated the blue light effect by regulating the expression of key genes in *A. flavus*; more details are necessary to clarify the function of LreA.

Our study identified a blue light sensor LreA and characterized the function of LreA in regulating conidiation and AFB1 biosynthesis in *A. flavus*, which suggested that blue light sensors may ubiquitously exist in Aspergillus fungi and play a role in reproduction growth and secondary metabolism. Our study advanced scientific knowledge and expanded the new research field of *A. flavus*, which is beneficial for the prevention of *A. flavus* and food safety.

## Figures and Tables

**Table 1:** The *Aspergillus flavus* strains used in this study.

Strain Name	Genotype	Source
Wild-type (WT)	Δ*ku70*, Δ*niaD*, Δ*pyrG*::*pyrG*	Preserved in this laboratory
CA14 PTS	∆*ku70*, Δ*niaD*, ∆*pyrG*	Purchased from FGSC
Δ*lreA*	Δ*ku70*, Δ*niaD*, ∆*pyrG*, Δ*lreA*::*pyrG*	This study
*lreA-com*	Δ*ku70*, Δ*niaD*, Δ*pyrG*::*lreA*, *pyrG*	This study
Δ*lreB*	Δ*ku70*, Δ*niaD*, ∆*pyrG*, Δ*lreB*::*pyrG*	This study
*lreB-com*	Δ*ku70*, Δ*niaD*, Δ*pyrG*::*lreB*, *pyrG*	This study
Δ*lreA*Δ*lreB*	Δ*ku70*, Δ*niaD*, ∆*pyrG*, Δ*lreA*::*pyrG*, *ΔlreB*::*ptrA*	This study
*lreA^ΔPAS-A^*	Δ*ku70*, Δ*niaD*, Δ*pyrG*::PAS-A, *pyrG*	This study

**Table 2:** The predicted functional partners of LreA.

Partners	Annotation
AFLA_065850	Sensor histidine kinase/response regulator (putative)
AFLA_051690	Cutinase gene palindrome-binding protein (putative)
velB	Velvet complex subunit B
LaeA	Secondary metabolism regulator laeA
AFLA_014710	Importin subunit alpha

## Data Availability

The original contributions presented in the study are included in the article, further inquiries can be directed to the corresponding author.

## Appendix A

**Table A1:** PCR primers used in this study.

Primer	Sequence (5′→3′)	Characteristics
*lreA*-a1	GGGGATGACTTGGACTGTGAC	For Δ*lreA* upstream homology arm
*lreA*-a3	GGGTGAAGAGCATTGTTTGAGGCCCCTTCCATGCTGTGGCTAAT	
*lreA*-b6	GCATCAGTGCCTCCTCTCAGACGCCACCGCCACTATTGTTA	For Δ*lreA* downstream homology arm
*lreA*-b8	TCTTCCCGCTTACCTTCCT	
*lreA*-a2	GATGCTGAAATTGGGACTGGTG	For Δ*lreA* overlap PCR
*lreA*-b7	GAGCTGGATATTCTGGTCAAGATGTA	
*lreA*-oF	CTCCGAAGCATTTGAACGACT	For Δ*lreA* test
*lreA*-oR	TTGTTGGCACCAGCTCTGTGA	
Q-*lrea*-F	CCACTCATCGCTTCATTCA	For *lreA* q-RT-PCR
Q-*lrea*-R	TTCGGTCTTGACACTACTTCG	
Co-A-a1	GCGAGCATAGGACACGGTAG	For *lreA-com* upstream homology arm
Co-A-a3	TAAAACCCTGGCTTCCCTCCTTCGCCTCAAACAATGCTCTTCACCC	
Co-A-b6	GCATCAGTGCCTCCTCTCAGACGAACAGCACTTAGGCCTG	For *lreA-com* downstream homology arm
Co-A-b8	CACATCCAGAGTCCCTTC	
Co-A-a2	ACTGGGTCGTATGCCTGATG	For *lreA-com* overlap
Co-A-b7	TCTTCCCGCTTACCTTCCTA	
*lreB*-A1	AAGGTTCACTCATAGGCCACA	For Δ*lreB* upstream homology arm
*lreB*-A3	GGGTGAAGAGCATTGTTTGAGGCGGAACTTCTCGTCCAATGCTG	
*lreB*-B6	GCATCAGTGCCTCCTCTCAGACGAAACATATTCCTGCGGCGATAC	For Δ*lreB* downstream homology arm
*lreB*-B8	TGCTGCTGCGAACCATAGACC	
*lreB*-A2	TGAGGCATAATCTGATCCAGC	For Δ*lreB* overlap
*lreB*-B7	CGAATAAAGATAACATCGGCTA	
CO-B-A1 CO-B-A3	TGGTTTCCCTGGTGCCAACGATGG TGAAGAGCATTGTTTGAGGCCTAGATTTGATCTTGCCGTTTC	For *lreB-com* upstream homology arm
CO-B-B6	CATCAGTGCCTCCTCTCAGACCCACCACCACCACCACTACGTGT	For *lre-comB* downstream homology arm
CO-B-B8	TGGCTTCAACAGTTCTCCC	
CO-B-A2	CGTCCGAACCGAACGACAG	For lreB-com overlap
CO-B-B7	CCGCCAGCACAGCAAGAGT	
Q-*lreB*-F	TACCGATTGCGGAACCTCT	For *lreB* q-RT PCR
Q-*lreB*-R	TTGATCTTGCCGTTTCTTT	
AB-A1	CTTCGTCTCCGTTCAACCT	For Δ*lreA*Δ*lreB* upstream homology arm
AB-A3	CAATTGCCCGTCTGTCAGATCCAACTTCTCGTCCAATGCTG	
AB-B6	GGCTCATCGTCACCCCATGATAGGCTCCGTCCTGCTATTTCT	For Δ*lreA*Δ*lreB* downstream homology arm
AB-B8	TGGCTTCAACAGTTCTCCC	
AB-B7	GATATTCTACCCGCCTGTG	For Δ*lreA*Δ*lreB* overlap
AB-A2	CTTCGTCTCCGTTCAACCT	
P1020-F	ATCGGCAATACCGTCCAGAAGC	For *pyrG* test
P801-R	CAGGAGTTCTCGGGTTGTCG	
*lreB*-OF	ATTGGACCGCTGCCGAACACG	For Δ*lreB* test
*lreB*-OR	ATGAGGCGACCCTGGCGAACAC	
*PAS*-A-A1	CGCTTGCTACGGCCCGCACTTGCATG	For lreA^ΔPAS-A^P1
*PAS*-A-A3	GCGCGAGGTGGTTTCGTCATCATTCGGTCTTGACACTACTTCG	
pyrG-F	GCCTCAAACAATGCTCTTCACCC	For *A. fumigatus pyrG*
pyrG-R	GTCTGAGAGGAGGCACTGATGC	
*PAS*-A-A4	GAAGTAGTGTCAAGACCGAATGATGACGAAACCACCTCGCGC	For *lreA*^Δ*PAS-A*^P2
*PAS*-A-A5	GAAGAGCATTGTTTGAGGCCGGTGGCTTTTTGGTTCTCTT	
*PAS*-A-B6	TCAGTGCCTCCTCTCAGACCCACTATTGTTATCATCATCAT	*For lreA* ^Δ*PAS-A*^ *P3*
*PAS*-A-B8	CAGGAGTTCTCGGGTTGTCG	
*PAS*-A-A2	TTCGGACTCATTCCATCT	For nested PCR
*PAS*-A-B7	CTCGGTGCCCATAAAGC	
*LOV*-F	ATAACGTCTGAAAGCTCCTCTG	For sequencing
*LOV*-R	TCAAACTTGGACCCTACTCG	

**Table A2:** Primers for qRT-PCR used in this study.

Primer	Primer Sequence (5′→3′)	Characteristics
*brlA*-F	GCCTCCAGCGTCAACCTTC	For amplifying *brlA*
*brlA*-R	TCTCTTCAAATGCTCTTGCCTC	
*abaA*-F	TCTTCGGTTGATGGATGATTTC	For amplifying *abaA*
*abaA*-R	CCGTTGGGAGGCTGGGT	
*aflR*-F	AAAGCACCCTGTCTTCCCTAAC	For amplifying *aflR*
*aflR*-R	GAAGAGGTGGGTCAGTGTTTGTAG	
*aflS*-F	AAGCTAAGGCCGAGTCTGG	For amplifying *aflS*
*aflS*-R	CAGGTTGTGTTGCTGTTGATAG	
*aflH*-F	TTCTTGCTCCTTGGTTCAT	For amplifying *aflH*
*aflH*-R	GGTCAAAGATTCCCTCGG	
*aflO*-F	GATTGGGATGTGGTCATGCGATT	For amplifying *aflO*
*aflO*-R	GCCTGGGTCCGAAGAATGC	
*aflJ*-F	CGGCGTATGAGGAGAATG	For amplifying *aflJ*
*aflJ*-R	CTTCATCAACCTGGCATCA	
*actin*-F	ACGGTGTCGTCACAAACTGG	For amplifying *actin*
*actin*-R	GCGTATCGTCGTTACCTCATC	
*q-LOV*-F	ATGTCGTACCGCCTCCTGAC	For amplifying *lreA*^ΔPAS-A^
*q-LOV*-R	TCCGCTGTATTTCTTTCGTATCCT	

## References

[1] Corrochano L.M. (2019). Light in the Fungal World: From Photoreception to Gene Transcription and Beyond. Annu. Rev. Genet..

[2] Yu Z., Fischer R. (2019). Light sensing and responses in fungi. Nat. Rev. Microbiol..

[3] Bayram O., Krappmann S., Ni M., Bok J.W., Helmstaedt K., Valerius O., Braus-Stromeyer S., Kwon N.J., Keller N.P., Yu J.H. (2008). VelB/VeA/LaeA complex coordinates light signal with fungal development and secondary metabolism. Science.

[4] Lee K., Loros J.J., Dunlap J.C. (2000). Interconnected feedback loops in the *Neurospora* circadian system. Science.

[5] Chen C.H., Ringelberg C.S., Gross R.H., Dunlap J.C., Loros J.J. (2009). Genome-wide analysis of light-inducible responses reveals hierarchical light signalling in *Neurospora*. Embo J..

[6] Purschwitz J., Muller S., Kastner C., Schoser M., Haas H., Espeso E.A., Atoui A., Calvo A.M., Fischer R. (2008). Functional and physical interaction of blue- and red-light sensors in *Aspergillus nidulans*. Curr. Biol..

[7] Zhang C., Selvaraj J.N., Yang Q., Liu Y. (2017). A Survey of Aflatoxin-Producing *Aspergillus* sp. from Peanut Field Soils in Four Agroecological Zones of China. Toxins.

[8] Yang M., Zhu Z., Zhuang Z., Bai Y., Wang S., Ge F. (2021). Proteogenomic Characterization of the Pathogenic Fungus *Aspergillus flavus* Reveals Novel Genes Involved in Aflatoxin Production. Mol. Cell Proteom..

[9] Cao W., Yu P., Yang K., Cao D. (2022). Aflatoxin B1: Metabolism, toxicology, and its involvement in oxidative stress and cancer development. Toxicol. Mech. Method..

[10] Dhakal A., Hashmi M.F., Sbar E. (2023). Aflatoxin Toxicity. StatPearls [Internet].

[11] Li Y., Meng X., Guo D., Gao J., Huang Q., Zhang J., Fischer R., Shen Q., Yu Z. (2022). A Simple and Low-Cost Strategy to Improve Conidial Yield and Stress Resistance of *Trichoderma guizhouense* through Optimizing Illumination Conditions. J. Fungi.

[12] Qin L., Li D., Zhao J., Yang G., Wang Y., Yang K., Tumukunde E., Wang S., Yuan J. (2021). The membrane mucin Msb2 regulates aflatoxin biosynthesis and pathogenicity in fungus *Aspergillus flavus*. Microb. Biotechnol..

[13] Jia K., Yan L., Jia Y., Xu S., Yan Z., Wang S. (2021). *aflN* Is Involved in the Biosynthesis of Aflatoxin and Conidiation in *Aspergillus flavus*. Toxins.

[14] Chang P.K., Scharfenstein L.L., Wei Q., Bhatnagar D. (2010). Development and refinement of a high-efficiency gene-targeting system for *Aspergillus flavus*. J. Microbiol. Meth.

[15] Jia K., Zhang D., Jia Q., Zhang Q.Y. (2019). Regulation of *Fgf15* expression in the intestine by glucocorticoid receptor. Mol. Med. Rep..

[16] Livak K.J., Schmittgen T.D. (2001). Analysis of relative gene expression data using real-time quantitative PCR and the 2(-Delta Delta C(T)) Method. Methods.

[17] Schmittgen T.D., Livak K.J. (2008). Analyzing real-time PCR data by the comparative C(T) method. Nat. Protoc..

[18] Cho H.J., Son S.H., Chen W., Son Y.E., Lee I., Yu J.H., Park H.S. (2022). Regulation of Conidiogenesis in *Aspergillus flavus*. Cells-Basel.

[19] Pfeifer A., Majerus T., Zikihara K., Matsuoka D., Tokutomi S., Heberle J., Kottke T. (2009). Time-Resolved Fourier Transform Infrared Study on Photoadduct Formation and Secondary Structural Changes within the Phototropin LOV Domain. Biophys. J..

[20] Fuller K.K., Ringelberg C.S., Loros J.J., Dunlap J.C. (2013). The fungal pathogen *Aspergillus fumigatus* regulates growth, metabolism, and stress resistance in response to light. mBio.

[21] Galindo L.J., Milner D.S., Gomes S.L., Richards T.A. (2022). A light-sensing system in the common ancestor of the fungi. Curr. Biol..

[22] Etxebeste O., Garzia A., Espeso E.A., Ugalde U. (2010). *Aspergillus nidulans* asexual development: Making the most of cellular modules. Trends Microbiol..

[23] Qin L., Yang L., Zhao J., Zeng W., Su M., Wang S., Yuan J. (2022). GTPase Rac Regulates Conidiation, AFB1 Production and Stress Response in Pathogenic Fungus *Aspergillus flavus*. Toxins.

[24] Park H.S., Yu J.H. (2016). Developmental regulators in *Aspergillus fumigatus*. J. Microbiol..

[25] Yu J.H. (2010). Regulation of Development in *Aspergillus nidulans* and *Aspergillus fumigatus*. Mycobiology.

[26] Adams T.H., Boylan M.T., Timberlake W.E. (1988). *brlA* is necessary and sufficient to direct conidiophore development in *Aspergillus nidulans*. Cell.

[27] Han S., Adams T.H. (2001). Complex control of the developmental regulatory locus *brlA* in *Aspergillus nidulans*. Mol. Genet. Genom..

[28] Son Y.E., Yu J.H., Park H.S. (2023). Regulators of the Asexual Life Cycle of *Aspergillus nidulans*. Cells.

[29] Georgianna D.R., Payne G.A. (2009). Genetic regulation of aflatoxin biosynthesis: From gene to genome. Fungal Genet. Biol..

[30] Yabe K., Nakajima H. (2004). Enzyme reactions and genes in aflatoxin biosynthesis. Appl. Microbiol. Biot..

[31] Caceres I., Khoury A.A., Khoury R.E., Lorber S., Oswald I.P., Khoury A.E., Atoui A., Puel O., Bailly J.D. (2020). Aflatoxin Biosynthesis and Genetic Regulation: A Review. Toxins.

[32] Heintz U., Meinhart A., Winkler A. (2014). Multi-PAS domain-mediated protein oligomerization of PpsR from Rhodobacter sphaeroides. Acta Crystallogr. D Biol. Crystallogr..

[33] Christie J.M., Blackwood L., Petersen J., Sullivan S. (2015). Plant flavoprotein photoreceptors. Plant Cell Physiol..

[34] Crosson S., Moffat K. (2002). Photoexcited structure of a plant photoreceptor domain reveals a light-driven molecular switch. Plant Cell.

[35] Dasgupta A., Fuller K.K., Dunlap J.C., Loros J.J. (2016). Seeing the world differently: Variability in the photosensory mechanisms of two model fungi. Env. Microbiol..

[36] Xu S.Y., Yu L., Luo X.C., Ying S.H., Feng M.G. (2023). Co-Regulatory Roles of WC1 and WC2 in Asexual Development and Photoreactivation of *Beauveria bassiana*. J. Fungi.

[37] Froehlich A.C., Liu Y., Loros J.J., Dunlap J.C. (2002). White Collar-1, a circadian blue light photoreceptor, binding to the frequency promoter. Science.

